# Sensitivity analysis of the CROPGRO-Canola model in China: A case study for rapeseed

**DOI:** 10.1371/journal.pone.0259929

**Published:** 2021-11-18

**Authors:** Mancan Xu, Chunmeng Wang, Lin Ling, William D. Batchelor, Jian Zhang, Jie Kuai

**Affiliations:** 1 Macro Agriculture Research Institute, College of Plant Science and Technology, Huazhong Agricultural University, Wuhan, Hubei Province, P.R. China; 2 Inspection and Quarantine Technology Communication Department, Shanghai Customs College, Shanghai, P.R. China; 3 Biosystems Engineering, Auburn University, Auburn, AL, United States of America; 4 Macro Agriculture Research Institute, College of Resources and Environment, Huazhong Agricultural University, Wuhan, Hubei Province, P.R. China; 5 College of Plant Science and Technology, Huazhong Agricultural University, Wuhan, Hubei Province, P.R. China; Julius Kuhn-Institut, GERMANY

## Abstract

Increasing domestic rapeseed production is an important national goal in China. Researchers often use tools such as crop models to determine optimum management practices for new varieties to increased production. The CROPGRO-Canola model has not been used to simulate rapeseed in China. The overall goal of this work was to identify key inputs to the CROPGRO-Canola model for calibration with limited datasets in the Yangtze River basin. First, we conducted a global sensitivity analysis to identify key genetic and soil inputs that have a large effect on simulated days to flowering, days to maturity, yield, above-ground biomass, and maximum leaf area index. The extended Fourier amplitude test method (EFAST) sensitivity analysis was performed for a single year at 8 locations in the Yangtze River basin (spatial analysis) and for seven years at a location in Wuhan, China (temporal analysis). The EFAST software was run for 4520 combinations of input parameters for each site and year, resulting in a sensitivity index for each input parameter. Parameters were ranked using the top-down concordance method to determine relative sensitivity. Results indicated that the model outputs of days to flowering, days to maturity, yield, above-ground biomass, and maximum leaf area index were most sensitive to parameters that affect the duration of critical growth periods, such as emergence to flowering, and temperature response to these stages, as well as parameters that affect total biomass at harvest. The five model outputs were also sensitive to several soil parameters, including drained upper and lower limit (SDUL and SLLL) and drainage rate (SLDR). The sensitivity of parameters was generally spatially and temporally stable. The results of the sensitivity analysis were used to calibrate and evaluate the model for a single rapeseed experiment in Wuhan, China. The model was calibrated using two seasons and evaluated using three seasons of data. Excellent nRMSE values were obtained for days to flowering (≤1.71%), days to maturity (≤ 1.48%), yield (≤ 9.96%), and above-ground biomass (≤ 9.63%). The results of this work can be used to guide researchers on model calibration and evaluation across the Yangtze River basin in China.

## Introduction

Rapeseed (*Brassica napus*) is the largest domestic source of vegetable oil in China, which accounts for 47% of the global supply [1]. Despite the current rise in domestic production of edible oil, a large percentage is still imported from other countries [2]. China is the largest global producer of rapeseed, with an annual production of approximately 16 MT and an average yield of approximately 1950 kg ha^-1^ in 2015 [3]. Rapeseed yield is primarily affected by nitrogen fertilizer, plant density and planting date [4, 5]. There are numerous studies on rapeseed response to different management practices [6, 7] and response mechanisms under abiotic stress [8]. However, there are few studies using crop models to optimize management strategies for rapeseed. Research on how to increase production under new management practices and genetics is needed to increase domestic production.

Crop growth models have been widely used around the world to study optimal field management practices, interactions between genetics and the environment, and develop strategies to mitigate the impact of climate change, leading to increased food production. Several widely used crop models simulate canola growth such as APSIM-Canola [9], DAISY-Rape [10], DSSAT-Rape [11], and EPIC-Rape [12]. WOFOST-GTC was developed to simulate the yield and oil quality of winter rapeseed [13]. HUME-OSR was developed and evaluated for winter rapeseed in Germany [14]. The APSIM-canola model has been evaluated in Australia, China, Germany, and America [9, 15, 16]. APSIM-canola was modified and used to simulate canola phenology and yield in China. The model accurately simulated canola phenology across different environments after correction and the influence of model uncertainty was reduced [17]. In a subsequent study, APSIM-Canola was used to investigate the impact of genotype, environmental and management practices on canola yield in different climatic regions of China. Results showed that in the upper and middle Yangtze River Basin regions where rainfall was sufficient, the simulated yield potential of winter canola was more than 4.8 t ha^-1^. However, in the northern region with limited rainfall, the yield potential was only 1.1 t ha^-1^ [18]. The model was also used to study the optimum water-use efficiency of canola production in Australia [19–21]. Tian et al. [22] coupled the APSIM-canola model with the AEZ model to simulate the potential production of canola in the Yangtze River Basin. The study simulated the impact of climate change on canola (winter rapeseed) in the Yangtze River Basin and showed that rape production in the Yangtze River Basin would increase in the 2050s. The average potential productivity of rapeseed in the upper, middle and lower reaches of the Yangtze River Basin was 0.939, 1.639 and 0.639 million tons respectively.

The CROPGRO-Canola model included in DSSAT 4.7 integrates the effect of weather, soil, management and genetic factors on the daily development and growth of canola and rapeseed [23–25]. Daily growth potential is computed based on photosynthetically active radiation (PAR) intercepted by the crop canopy. Growth is modified based on temperature and water and nitrogen stress factors [26]. Model input data includes daily weather (max/min temperature, rainfall, solar radiation), soil properties, cultivar coefficients and management practices. The model has been tested for canola in Italy [27], Canada [28] and Pakistan [29]. However, the model has not been tested for rapeseed in China.

A sensitivity analysis (SA) is often used to aid in the calibration and evaluation of a model in a new region. Global sensitivity analysis identifies important model inputs that should be measured or calibrated and facilitates calibration and evaluation of models [30]. In addition, global sensitivity analysis can be used to effectively evaluate the temporal and spatial stability of model inputs. There are several global sensitivity methods including Morris [31], regression [32], Sobol [33], and the extended Fourier amplitude test method (EFAST) [34]. The EFAST method incorporates advantages from both the Sobol and the Fourier amplitude test (FAST methods) [34] and is based on the idea of variance decomposition. In this approach, a sensitivity index (SI) of each parameter is obtained through probability density function conversion, Fourier transformation, and variance decomposition. This method offers the advantages of high calculation accuracy and requires only a few samples to study the influence of multiple parameters on the model output results [35, 36].

The overall goal of this work was to conduct a spatial and temporal sensitivity analysis of the CROPGRO-Canola model for rapeseed in the Yangtze River Valley and to use the sensitivity analysis to guide model calibration and evaluation of a simple rapeseed experiment. The specific objectives were to:

1. use the EFAST method to evaluate the spatial and temporal sensitivity of important model inputs on key model outputs;
2. use the top-down concordance coefficient index to evaluate the consistency of the sensitivity results across different years and locations;
3. conduct calibration and evaluation of the model for a simple rapeseed experiment.

## Materials and methods

### Site data for spatial and temporal sensitivity analysis

A sensitivity analysis to evaluate the spatial sensitivity of input parameters was performed using a combination of rapeseed experiments from eight sites in the Yangtze River basin, including data from 2011 to 2018 (Table 1). Management information for each experiment was used as model inputs. The site in Wuhan consisted of seven years of data and was used to evaluate the temporal sensitivity of input parameters, while the remainder of the experiments consisted of one season of data and were used to evaluate the spatial sensitivity of input parameters. These sites represented a range in rainfall (221–959 mm), mean temperature (10.4–17.4°C) and solar radiation (1708–2954 MJ m^-2^ d^-1^). The management practices for each site are shown in Table 2.

**Table 1:** Site information and meteorological summary of the rapeseed growing season.

Site	Latitude	Longitude	Altitude	Tm	Rain	Radiation	Experimental period
(Province)	(°)	(°)	(m)	(°C)	(mm)	(MJ m^-2^ d^-1^)	
Luxi	24.32	103.46	1704.3	15.0	240.2	2954.3	2018
(Yunnan)							
Chongqing	29.35	106.28	259.1	14.4	411.7	1718.4	2018
Hanzhong	33.04	107.02	509.5	10.4	221.2	1976.7	2018
(Shanxi)							
Zhuzhou	27.52	113.10	74.6	12.5	958.7	1791.6	2018
(Hunan)							
Wuhu	31.09	118.35	17.1	11.1	572.8	2090.2	2018
(Anhui)							
Gaoyou	32.48	119.27	5.4	10.5	354.8	2365.0	2018
(Jiangsu)							
Hangzhou	30.14	120.10	41.7	12.4	839.9	2040.5	2018
(Zhejiang)							
Wuhan	30.47	114.35	23.6	17.4	530.6	2162.4	2011–2018
(Hubei)							

**Table 2:** Management practices at eight sites.

Site (Province)	Soil type	Planting date	Row spacing (cm)	Population (plants m^-2^)	N rate (kg ha^-1^)
Luxi	Red soil	09/20/2018	25	45	174
(Yunnan)					
Chongqing	Red soil	09/28/2018	25	45	174
Hanzhong	Paddy soil	09/26/2018	25	45	174
(Shanxi)					
Zhuzhou	Yellow brown soil	09/28/2018	25	45	174
(Hunan)					
Wuhu	Paddy soil	10/01/2018	25	45	174
(Anhui)					
Gaoyou	Paddy soil	10/03/2018	25	45	174
(Jiangsu)					
Hangzhou	Red soil	10/05/2018	25	45	174
(Zhejiang)					
Wuhan	Yellow brown soil	09/28/2018	25	45	174
(Hubei)					

### The CROPGRO-Canola model

Daily weather data needed to run the CROPGRO-Canola model were taken from the National Meteorological Science Data Center database (http://data.cma.cn/), which included daily maximum and minimum temperature, precipitation, and sunshine hours. Daily solar radiation needed by the model was computed using the Angstrom equation,

$$R_{s}=(a+b\frac{n}{N}R_{a})$$ (1)

where Rs is total solar radiation in MJ m^-2^, Ra is Astronomical radiation in MJ m^-2^, n is measured solar sunshine hours in h, N is theoretical sunshine hours in h and a and b are coefficients (a = 0.2, b = 0.5) [37].

The soil information came from the China soil data set in the Harmonized World Soil Database (HWSD). The variety Huayouza 62, one of the main winter rapeseed varieties in the Yangtze River Basin, was used as the default variety. The genetic coefficients for this variety were set as default in the model, and then adjust after sensitivity analysis to match experimental data for the case study.

Three types of model input parameters, genotype and ecotype coefficients, and soil parameters, were selected for the sensitivity analysis. Model outputs selected for the sensitivity analysis included days to flowering and days to maturity, yield, above-ground biomass at harvest and maximum leaf area index. Parameter abbreviations, definitions and value ranges are shown in Table 3. The range for genotype parameters was taken from recommendations from the Glue cultivar coefficient optimizer tool in DSSAT. The range for ecotype parameters was ±30% of the value for the cultivar selected for this study. The range of soil parameters was based on expected ranges from the literature [38]. Other management practice inputs needed for the model runs were taken from the experiments conducted at each site.

**Table 3:** Selected parameters and output variables in CROPGRO-Canola model.

Codes	Definitions	Range
Canola Ecotype Parameters		
PL-EM	Time between planting and emergence/ thermal days	2.52–4.68
EM-V1	Time required from emergence to first true leaf/ thermal days	4.2–7.8
JU-R0	Time required for floral induction, equal to the minimum number of days for floral induction under optimal temperature and daylengths/ thermal days	3.5–6.5
PM09	Proportion of time between first seed and physiological maturity that the last seed can be formed	0.25–0.46
LNGSH	Time required for growth of individual shells/ thermal days	7–13
R7-R8	Time between physiological and harvest maturity / thermal days	8.4–15.6
TRIFL	Rate of appearance of leaves on the mainstem	0.22–0.42
RWDTH	Relative width of this ecotype in comparison to the standard width per node (YVSWH) defined in the species file	0.7–1.3
RHGHT	Relative height of this ecotype in comparison to the standard height per node (YVSHT) defined in the species file	0.63–1.17
R1PPO	Increase in daylength sensitivity after flower appearance/ h	0.35–0.66
OPTBI	Minimum daily temperature above which there is no effect on slowing normal development toward flowering/°C	14–26
SLOBI	Slope of relationship reducing progress toward flowering if TMIN for the day is less than OPTBI	0.02–0.05
Canola Genotype Parameters		
CSDL	Critical short daylength below which reproductive development progresses with no daylength effect/ h	14~24
EM-FL	Time between plant emergence and flower appearance/ photothermal days	20~45
FL-SH	Time between first flower and first pod/ photothermal days	10~16
FL-SD	Time between first flower and first seed/ photothermal days	15~35
SD-PM	Time between first seed and physiological maturity/ photothermal days	20~40
FL-LF	Time between first flower and end of leaf expansion/ photothermal days	1~10
SLAVR	Specific leaf area of cultivar under standard growth conditions/ (cm^2^g^-1^)	200~275
SIZLF	Maximum size of full leaf/ cm^2^	90~110
SFDUR	Seed filling duration for pod cohort at standard growth conditions/photothermal days	18~22
SDPDV	Average seed per pod under standard growing conditions	15~25
PODUR	Time required for cultivar to reach final pod load under optimal conditions/ photothermal days	8~12
Soil Parameters		
SLLL	Lower limit/(cm^3^cm^-^^3^)	0.055~0.123
SDUL	Upper limit/(cm^3^cm^-^^3^)	0.123~0.348
SSAT	Upper limit, saturated/(cm^3^cm^-^^3^)	0.348~0.547
SRGF	Root growth factor	0.7~1.0
SSKS	Saturated hydraulic conductivity/(cm h^-1^)	0.06~21.00
SBDM	Bulk density/(g cm^-^^3^)	1.0~1.4
SLOC	Organic carbon content/%	0.348~5.00
SALB	Albedo	0.09~0.17
SLU1	Evaporation limit/mm	2~12
SLDR	Drainage rate	0.01~0.85
SLRO	Runoff curve number	61~94
SLNF	Mineralization factor	0~1
	Output Variable	
ADAP	Anthesis day	
MDAP	Maturity day	
HWAM	Yield at harvest	
CWAM	Aboveground biomass at maturity	
LAIX	Maximum leaf area index	

### EFAST sensitivity analysis method

The EFAST method employs variance decomposition to perform a quantitative global sensitivity analysis of the objective function. Consequently, it uses variance to relate changes in model outputs to changes in model inputs. Previous studies have adopted this method to reflect the importance of the parameter and the degree of contribution to model result [38–40]. Total variance of the model was calculated by:

$$V(Y)=\sum_{i=1}^{m}V_{i}+\sum_{1\leq i\leq j\leq m}V_{ij}+\sum_{1\leq i\leq j\leq m}V_{1,2,\ldots ,m}$$ (2)

Where Vi was the variance of input parameter Xi, and Vij~V1, 2,…, m was the variance of the interaction among each parameter.

Model output variance, Vi, caused by a change in parameter Xi was calculated by:

$$V_{i}=V[E(Y/X_{i})]$$ (3)

The model output variance caused by the interaction of parameters Xi and Xj, was obtained using:

$$V_{ij}=V[E(Y/X_{i},X_{j})]-V_{i}-V_{j}$$ (4)

The first-order sensitivity index, Si, of parameter Xi reflected the contribution of parameter Xi to the total variance of model output parameters, as by:

$$S_{i}=V_{i}/V(Y)$$ (5)

The interaction of parameter Xi on the total variance of the model results and the interaction with other parameters was expressed by S_Ti_:

$$S_{Ti}=\left[V(Y)-V_{-i}\right]/V(Y)$$ (6)

Where V_-i_ is the total variance of all parameters except parameter Xi.

The values Si and S_Ti_ are constants, ranging between 0–1, with large values indicating greater influence of the input parameter on the output parameter. Larger values indicate that the model output is very sensitive to the input parameter being evaluated.

### Spatial and temporal sensitivity analysis

We used the EFAST method to evaluate the impact of 35 input parameters on 5 output parameters including days to anthesis and maturity, yield, above-ground biomass, and maximum leaf area index to assess model sensitivity for the sites shown in Table 1. The 35 input parameters (Table 3) included 12 ecotypes, 11 genotypes and 12 soil parameters. The spatial sensitivity analysis was run for one year (2018) using weather, soil and management information from 7 sites that represent a primary rapeseed production region in China (Table 1). Next, we ran the analysis for 7 years at one site (Wuhan) to assess the temporal sensitivity of model parameters using model input data collected from a long-term experiment.

We calculated the global sensitivity index using the SimLab (ver.2.2.1) Software, based on 4520 groups of random parameters in the set of parameters generated using a Monte Carlo sampling distribution. An R program was used for batch processing of the CROPGRO model results. Finally, we computed the first order and total sensitivity indices of the outputs to each set of input parameters using the SimLab Software.

The top-down concordance coefficient (TDCC) [41] method was used to evaluate the ranking of the sensitivity index of each parameter to determine the spatial and temporal consistency of sensitivity analysis results in this study. First, we defined the savage score of each parameter as follows:

$$S_{ij}=\sum_{i=r_{ij}}^{n}1/i$$ (7)

where, *S*_*ij*_ is the savage score of parameters *X*_*i*_ in replicates *R*_*j*_; r_ij_ is the ranking of *X*_*i*_ in replicates *R*_*j*_; and *n* is the number of parameters.

For all the different replicates, the TDCC was calculated using the following formula:

$$TDCC=\frac{\sum_{i=1}^{n}{\left(\sum_{j=1}^{m}S_{ij}\right)}^{2}-m^{2}n}{m^{2}(n-\sum_{i=1}^{n}1/i)}$$ (8)

where, *m* is the number of sensitivity analysis replicates.

The corresponding *p*-values of TDCC were calculated using the T statistics (approximating a *x*^2^-distribution with n-1degrees of freedom) as follows:

T=m⋅(n−1)⋅TDCC (9)

A small TDCC value (closer to 1 with a *p*-value less than 0.05) indicated that the results of each replicate were significantly consistent, and vice versa [42].

### Calibration and evaluation of the model based on sensitivity analysis results

A 5-year experiment collected from the literature [43–45] was used to calibrate and evaluate the CROPGRO-Canola model for winter rapeseed based on the sensitivity analysis results. The experiments for the calibration (2014, 2015) and evaluation (2016, 2017, and 2018) using the Huayouza 62 variety were conducted at a research site at Huazhong Agricultural University, Hubei Province. The datasets (Table 4) included management strategy, phenology, yield, and above-ground biomass of each experiment.

**Table 4:** Datasets for calibration (1–2) and evaluation (3–5) of CROPGRO-Canola model.

Data set	Sowing date	Fertilizer (kg ha^-1^)	Population (plants m^-2^)	Anthesis date	Maturity date	Measured yield (kg ha^-1^)	Measured above-ground biomass (kg ha^-1^)
1^[^^43^^]^	2014/9/25	N240, P66, K124	45	2015/2/22	2015/4/28	2648	13293
2^[^^44^^]^	2015/9/24	N199, P49, K93	45	2016/2/20	2016/4/22	2676	9757
3^[^^44^^]^	2016/9/27	N199, P49, K93	45	2017/2/14	2017/4/25	2641	9394
4^[^^45^^]^	2017/9/25	N139, P40, K75	45	2018/3/2	2018/4/30	2929	12467
5^[^^45^^]^	2018/9/27	N139, P40, K75	45	2019/3/4	2019/5/1	2765	12328

We used two years of data to calibrate (2014, 2015) and three years to evaluate the model (2016, 2017, and 2018). The most sensitive input parameters found in the sensitivity analysis were used for model calibration. Calibration was performed using a “trial and error” method to adjust input parameters to minimize the error between simulated and measured flowering date, maturity date, yield and above-ground biomass at harvest. Calibrated parameters were then used to evaluate the model performance in 2016, 2017, and 2018. The soil information came from the China soil data set in the Harmonized World Soil Database (HWSD) (Table 5).

**Table 5:** Physical and chemical properties of the soil.

Soil depth(cm)	Clay content (%)	Silt content (%)	Permanent wilting point (cm^3^ cm^-3^)	Field capacity (cm^3^ cm^-3^)	Saturated hydraulic conductivity (cm^3^ cm^-3^)	Root growth factor, soil only (0–1)	pH	Organic carbon content (%)	Bulk density (g cm^-3^)
5	21	50	0.153	0.34	0.447	1	7.8	1.12	1.22
15	21	50	0.153	0.34	0.447	1	7.8	1.12	1.22
30	21	50	0.153	0.34	0.447	0.7	7.8	1.12	1.22
60	21	45	0.144	0.314	0.414	0.2	7.9	0.82	1.31
80	21	45	0.144	0.314	0.414	0.05	7.9	0.82	1.31
100	21	45	0.144	0.314	0.414	0.03	7.9	0.82	1.31
120	21	45	0.144	0.314	0.414	0.03	7.9	0.82	1.31
150	21	45	0.144	0.314	0.414	0.03	7.9	0.82	1.31
180	21	45	0.144	0.314	0.414	0.03	7.9	0.82	1.31
200	21	45	0.144	0.314	0.414	0.03	7.9	0.82	1.31

## Results

### Temporal sensitivity analysis

**Fig 1:**
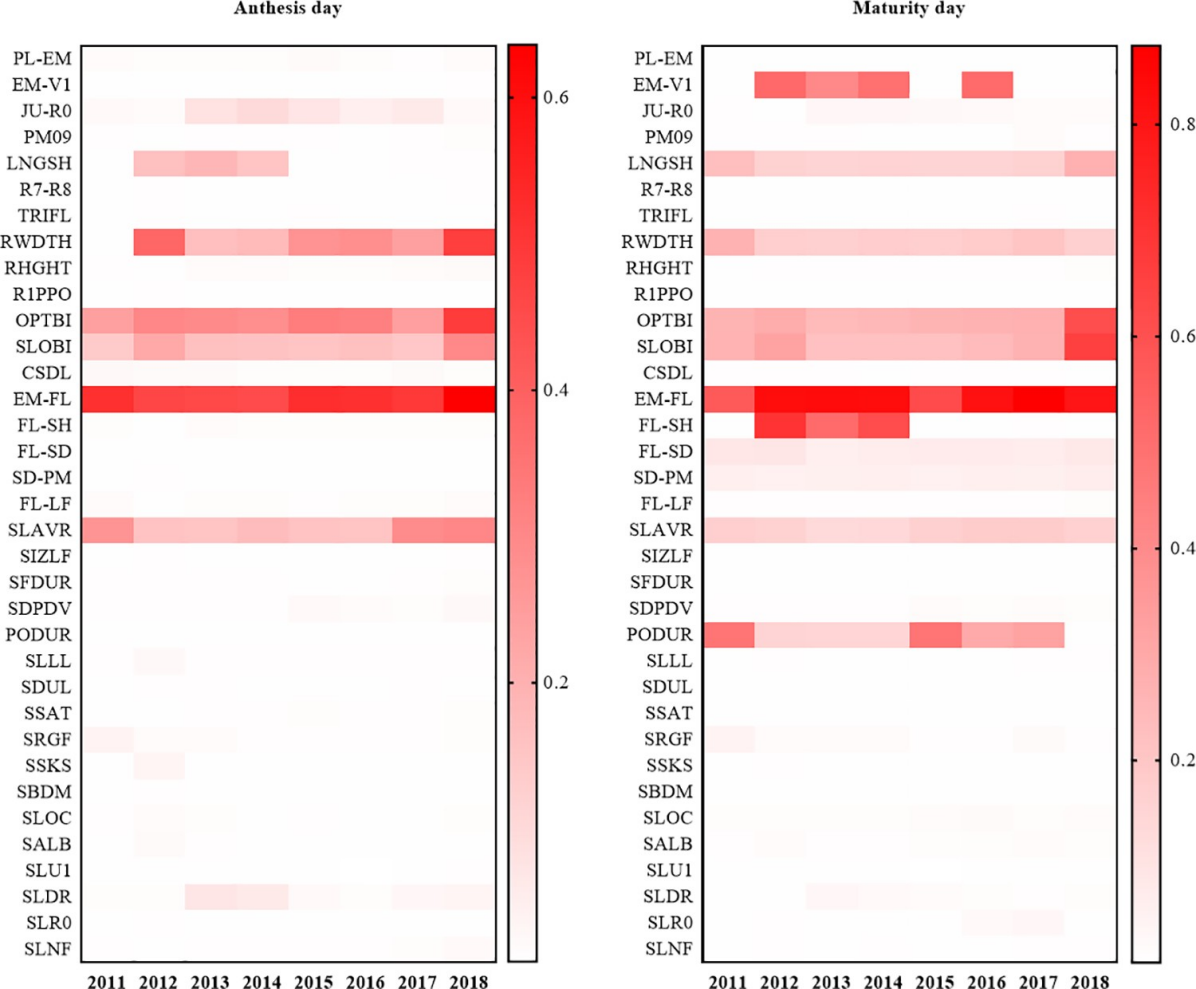
Temporal sensitivity indices for days to flowering and maturity (2011–2018). *Note*. I: ecotype parameters; II: genotype parameters; III: soil parameters.

The sensitivity analysis (Fig 1) showed that simulated days to flowering at the Wuhan site (2011 to 2018) were most sensitive to five primary input parameters. These included (i) EM-FL, described as photo-thermal days (PTD) between plant emergence and flower appearance, (ii) OPTBI, or minimum daily temperature above which there is no effect on slowing normal development toward flowering, (iii) RWDTH, canopy width of this ecotype in comparison to the standard width per node (YVSWH), (iv) SLAVR, which is specific leaf area of the cultivar under standard growth conditions, and (v) SLOBI which is the slope of the relationship reducing progress toward flowering if TMIN for the day is less than OPTBI. The average sensitivity indices were 0.50 for EM-FL, 0.31 for OPTBI, 0.24 for RWDTH, 0.21 for SLAVR, and 0.18 for SLOBI.

**Fig 2:**
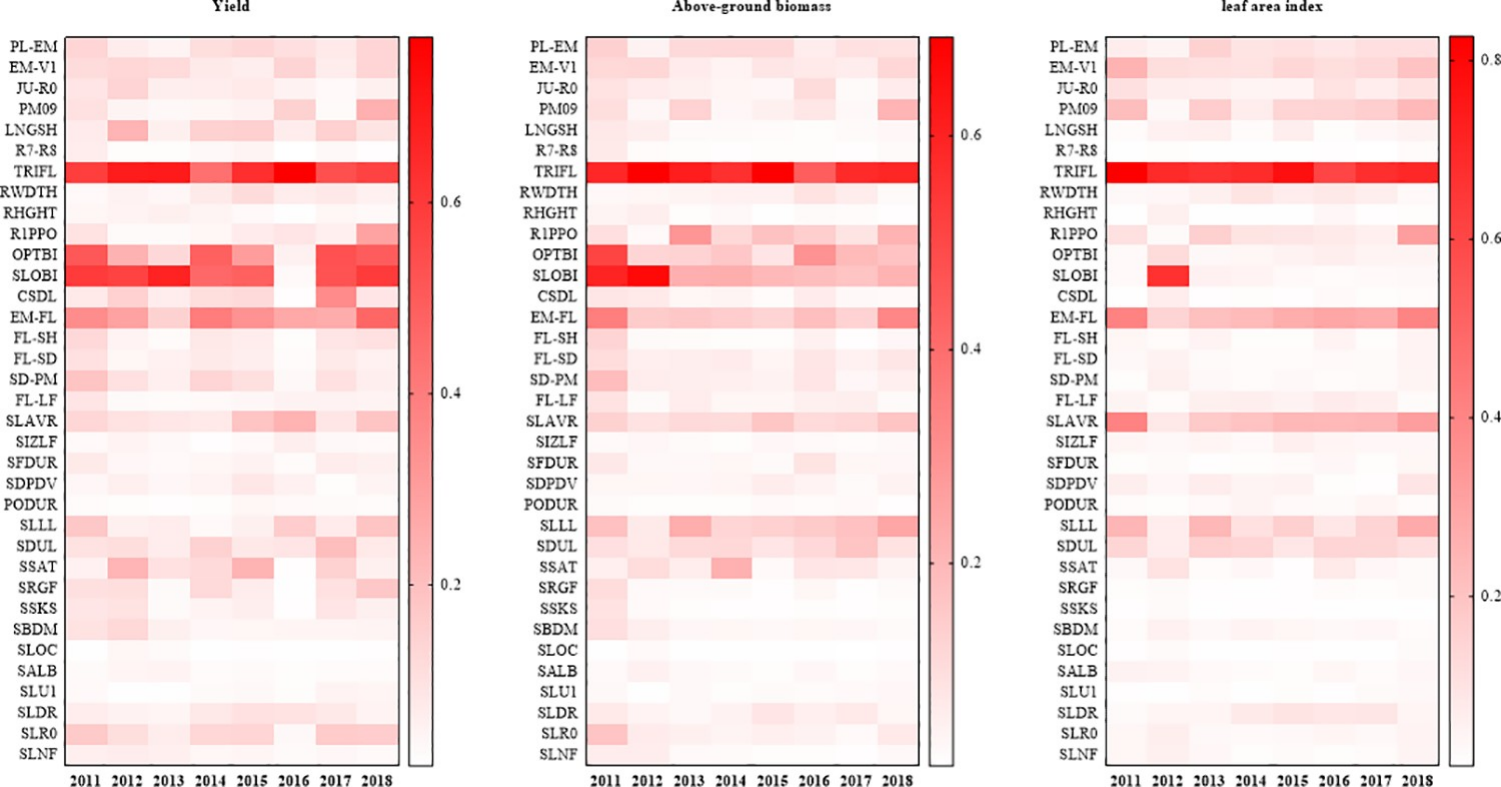
Temporal sensitivity indices for yield, aboveground biomass and LAI (2011–2018). *Note*. I: ecotype parameters; II: genotype parameters; III: soil parameters.

Days to maturity were most sensitive to the EM-FL parameter, with an average sensitivity index of 0.77. Days to maturity were also sensitive to OPTBI, SLOBI, PODUR (PTD required for a cultivar to reach final pod load under optimal conditions), EM-V1 (PTD required from emergence to first true leaf), and FL-SH (PTD between first flower and first pod). The average sensitivity indices were 0.306 for OPTBI, 0.300 for SLOBI, 0.255 for PODUR, 0.246 for EM-V1, and 0.235 for FL-SH. In addition, days to maturity were also sensitive to RWDTH, LNGSH (PTD required for growth of individual shells), and SLAVR with sensitivity indices of 0.19, 0.18, and 0.16, respectively. Conversely, soil parameters had little effect on phenology across the multi-year simulation (Figs 1 and 2) because development is primarily driven by daylength and temperature.

Fig 2 shows the sensitivity indices of 35 input variables on yield, above-ground biomass and LAI. The yield was most sensitive to TRIFL (rate of appearance of leaves on the mainstem), with a sensitivity index of 0.61, followed by SLOBI (SI = 0.49), OPTBI (SI = 0.34) and EM-FL (SI = 0.31). Some parameters had smaller sensitivity indices, including SLAVR, CSDL (critical short-day length below which reproductive development progresses with no daylength effect), SLR0 (runoff curve number), SSAT (Upper limit, saturated), LNGSH, SDUL (upper limit), EM-V1. Their sensitivity indices ranged from 0.10 to 0.15. The yield was sensitive to soil parameters that influenced water holding capacity and seasonal water stress (SLRO, SSAT, SDUL).

TRIFL had the greatest impact on aboveground biomass, with a sensitivity index of 0.59, followed by SLOBI (SI = 0.31) and OPTBI (SI = 0.21). Aboveground biomass was moderately sensitive to EM-FL, SLLL, R1PPO (increase in daylength sensitivity after flower appearance), SLAVR, and SDUL. Their sensitivity indices ranged from 0.10–0.20. Other parameters (SLOBI, TRIFL, OPTBI, EM-FL, SD-PM, SLLL, FL-SH, SCEC, SLAVR, SLR0, PL-EM, EM-V1, SRGF) also affected aboveground biomass in 2011 (Fig 2), possibly because Wuhan received less total rainfall (407.5 mm) between October 2011 and May 2012. These findings indicate that soil factors can have a significant impact on rapeseed growth.

LAI was most sensitive to TRIFL, which had a sensitivity index of 0.70, followed by EM-FL (SI = 0.28), SLAVR (SI = 0.24), and SLLL (SI = 0.16). The parameters PM09 (proportion of time between first seed and physiological maturity that the last seed can be formed), EM-V1, SDUL, R1PPO, and SLOBI had relatively low sensitivity indices, ranging from 0.10–0.15.

### Spatial sensitivity analysis

**Fig 3:**
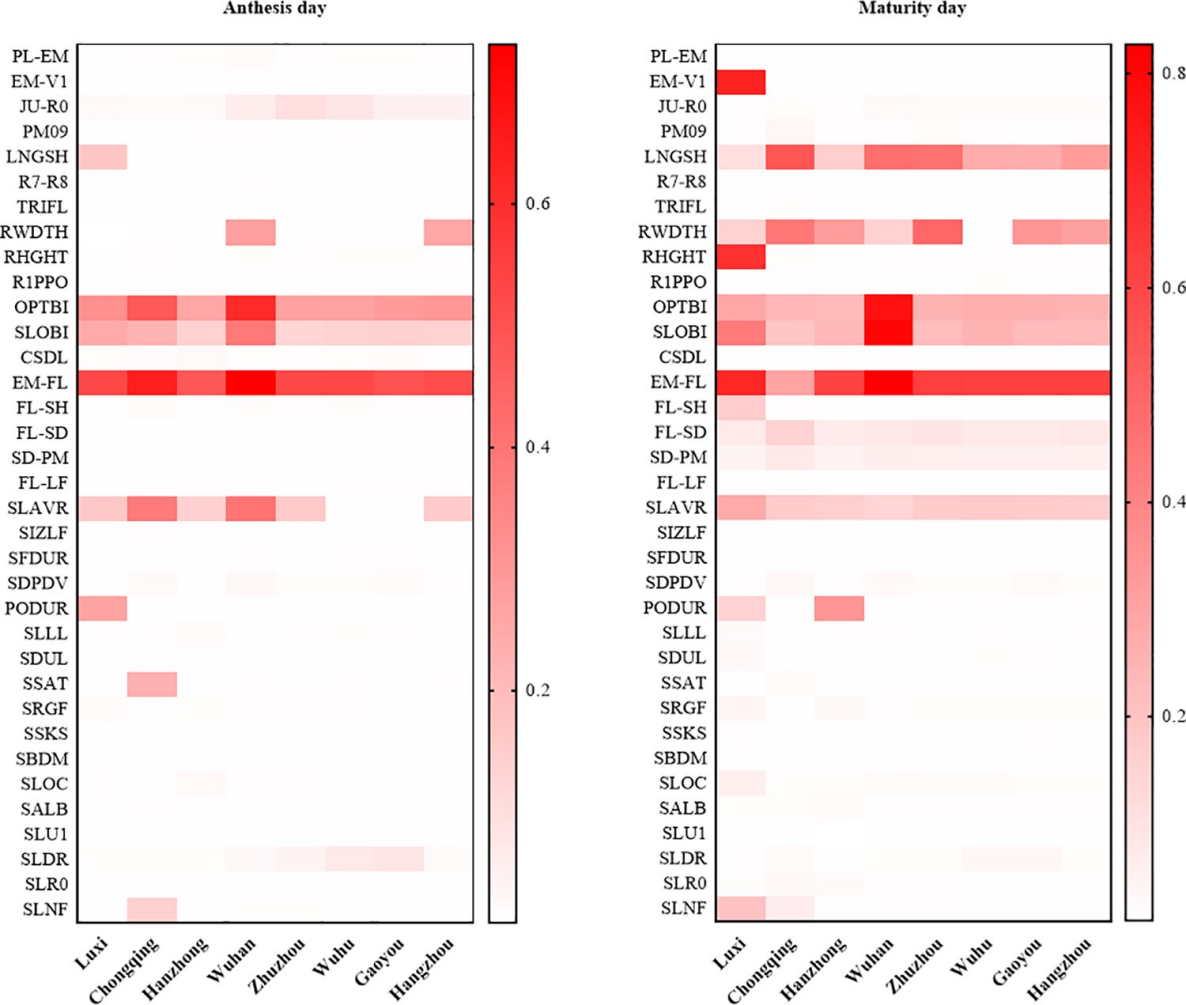
Spatial sensitivity indices of phenology to model input parameters. *Note*. I: ecotype parameters; II: genotype parameters; III: soil parameters.

Profiles of the sensitivity of days to flowering to model input parameters using weather and soil information at eight locations are shown in Fig 3. Days to flowering were most sensitive to EM-FL (SI = 0.55), OPTBI (SI = 0.35), SLOBI (SI = 0.19), and SLAVR (SI = 0.18). Days to maturity were most sensitive to EM-FL, LNGSH, SLOBI, OPTBI, and RWDTH. Parameters that strongly impact days to flowering often exhibited a greater impact on days to maturity, although some only affected the duration of time between flowering and maturity. Flowering and maturity date were not sensitive to soil parameters. This was expected since soil parameters (SLLL, SDUL, SSKS) impact water holding capacity and drought stress, and drought stress does not impact development to a large degree in the crop model.

**Fig 4:**
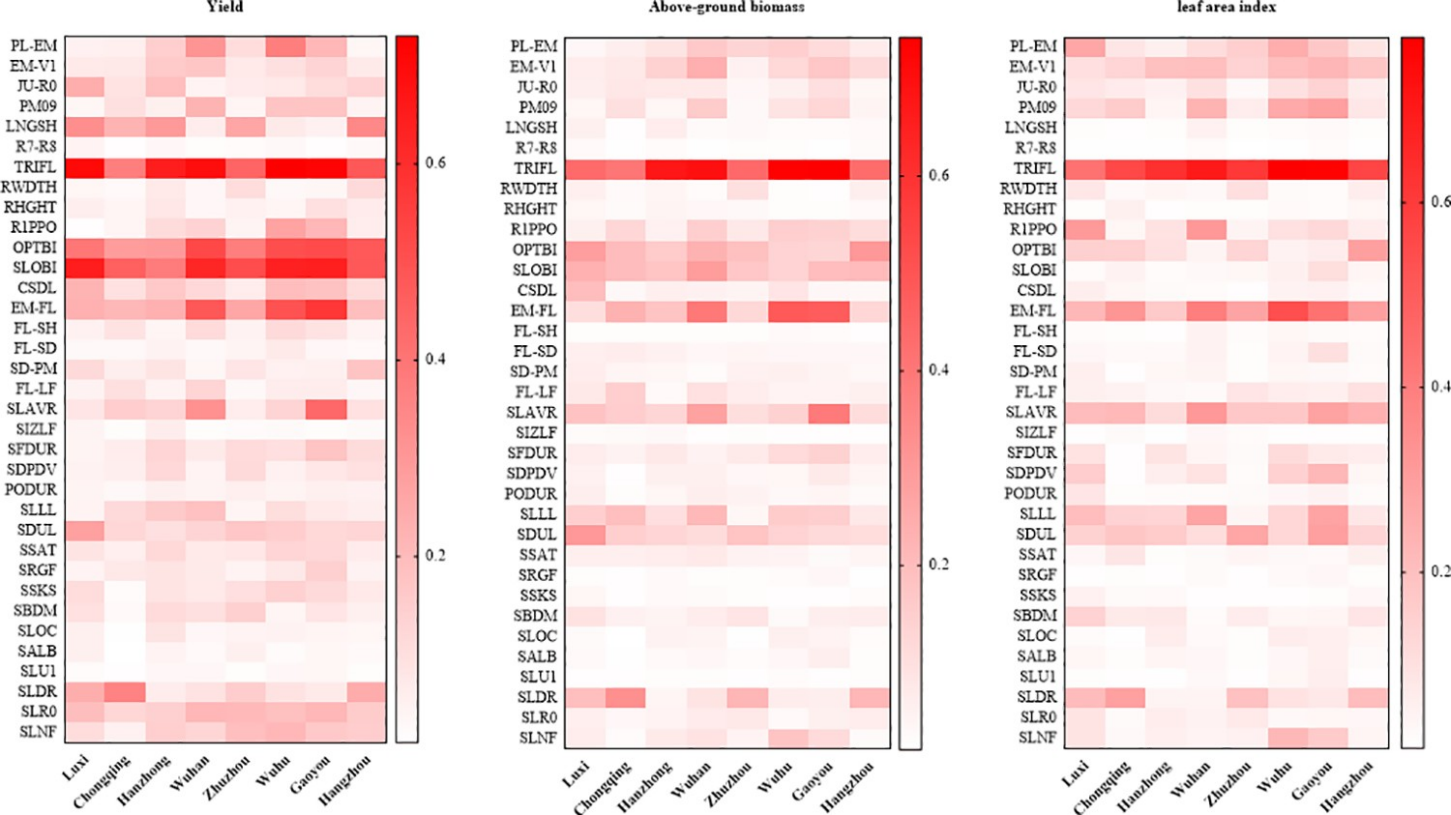
Spatial sensitivity indices of yield, aboveground biomass and LAI to input parameters. *Note*. I: ecotype parameters; II: genotype parameters; III: soil parameters.

The sensitivity of yield to input parameters varied spatially (Fig 4). Parameters that had the largest sensitivity indices for yield included TRIFL (SI = 0.6), SLOBI (SI = 0.55), OPTBI (SI = 0.42), EM-FL (SI = 0.33), and LNGSH (SI = 0.20). The input parameters SLR0, SLAVR, SLDR, PL-EM (time between planting and emergence) and other parameters also impacted yield differently at the different locations. Parameters that had the most impact on aboveground biomass included TRIFL (SI = 0.57), EM-FL (SI = 0.26), OPTBI (SI = 0.20), and SLOBI (SI = 0.20. Above-ground biomass was also sensitive to other parameters including SLAVR, SLDR, and SLLL, with SI ranging between 0.1–0.2. The main parameters affecting LAI were associated with the growth of leaves such as TRIFL (SI = 0.63), EM-FL (SI = 0.32) and SLAVR (SI = 0.22). Soil parameters including SDUL, SLLL and SLDR had less effect on maximum LAI and the sensitivity indices ranged between 0.1 and 0.2.

### Consistency analysis of each sensitivity test

A summary of consistency indices and significance of the sensitivity analysis over multiple years and sites is shown in Table 6. Both temporal and spatial tests showed which input parameters had the largest effect on development and growth, indicated by the top-down concordance coefficient and probability level. These findings indicate that there is both spatial and temporal consistency in parameters that have the largest influence on growth and development. This means that the parameters are important for calibration, independent of location or year. Key parameters which influenced days to flowering, days to maturity, yield, above-ground biomass, and the maximum leaf area index most were consistent in this area under different meteorological conditions.

**Table 6:** Top-Down Concordance Coefficients (TDCC) and related p-values obtained from each sensitivity analysis experiment.

	Temporal stability		Spatial stability		Total	
	TDCC	p-value	TDCC	p-value	TDCC	p-value
ADAP	0.870	<0.001	0.827	<0.001	0.817	<0.001
MDAP	0.821	<0.001	0.784	<0.001	0.767	<0.001
HWAM	0.744	<0.001	0.792	<0.001	0.738	<0.001
CWAM	0.839	<0.001	0.811	<0.001	0.790	<0.001
LAI	0.843	<0.001	0.823	<0.001	0.813	<0.001

### Calibration and evaluation of CROPGRO-Canola model in China

**Fig 5:**
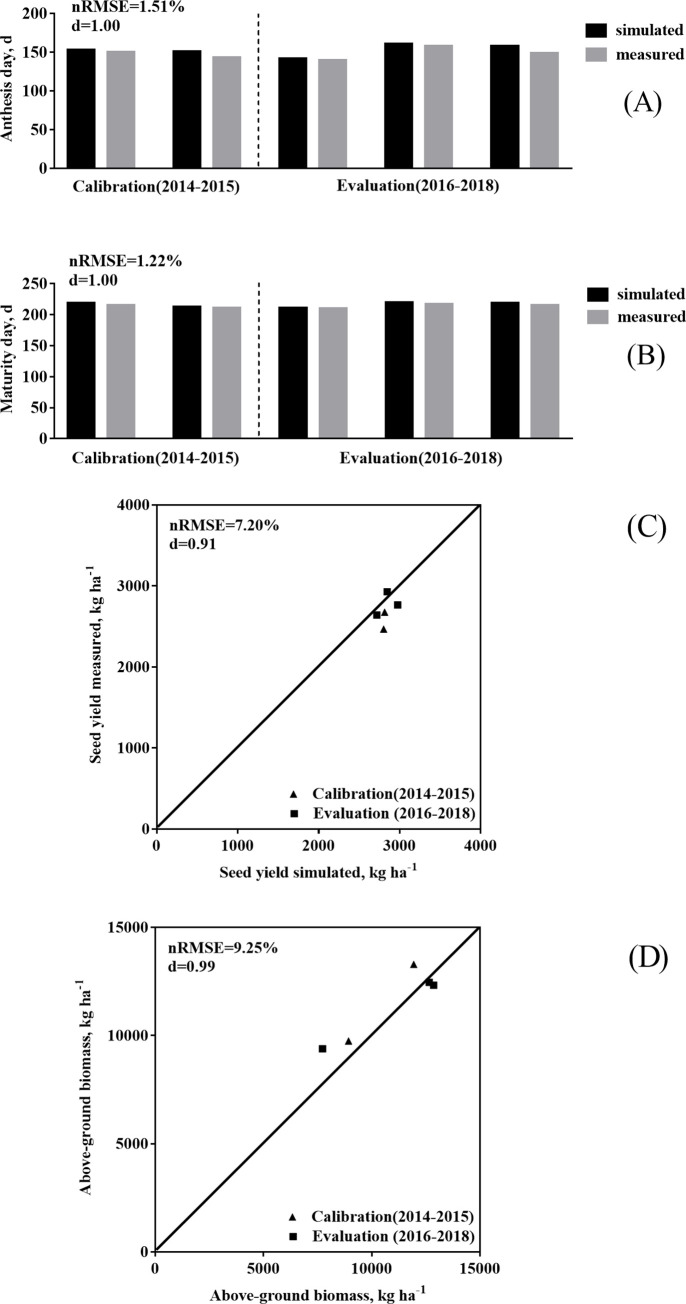
Results of calibration and evaluation for phenology (A) (B), yield (C) and above-ground biomass (D).

The model was calibrated for two seasons with limited growth and development data at Wuhan, China (2014, 2015) based on the results from the sensitivity analysis. The parameters that had the most influence on development and growth were calibrated using a “trial and error” method to minimize the error between simulated and observed values. Other parameters were fixed after slight adjustment. Results (Table 7) showed that flowering and maturity dates of rapeseed could be effectively calibrated by adjusting EM-FL, OPTBI and SLOBI. In addition, adjusting parameters such as TRIFL and SLAVR gave an excellent simulation of rapeseed yield and above-ground biomass. Results of model calibration and evaluation based on the sensitivity analysis and corresponding statistical indicators are shown in Fig 5 and Table 8. In conclusion, the nRMSE of flowering day, maturity day, yield and above-ground biomass for the calibration years were less than 1.71%, 1.48%, 9.96% and 9.63%, respectively. The nRMSE for flowering day, maturity day, yield and above-ground biomass for the evaluation years were 1.37%, 1.01%, 4.94% and 8.91%, respectively. The model efficiency (d-statistic) values were all high, indicating a good model fit.

**Table 7:** Calibrated cultivar coefficients for the most sensitive inputs.

Parameters	Default value	Calibrated value
EM-FL	29	39.45
OPTBI	20	7.5
SLOBI	0.035	0.064
SLAVR	250	319.2
TRIFL	0.32	0.23

**Table 8:** Statistical indicators for calibration and evaluation of the model.

Attribute	Statistical indicators					
	N	RMSE	nRMSE (%)	ME	rME (%)	d
	Calibration years (2014, 2015)					
Anthesis day, d	2	2.55	1.71	2.50	1.67	0.99
Maturity day, d	2	3.16	1.48	3.00	1.41	0.99
Seed yield, kg ha^-1^	2	256.19	9.96	236.50	9.20	0.72
Above-ground biomass, kg ha^-1^	2	1109.87	9.63	-1075.00	-9.33	0.99
	Evaluation years (2016, 2017, and 2018)					
Anthesis day, d	3	2.08	1.37	1.67	1.10	1.00
Maturity day, d	3	2.16	1.01	2.00	0.93	1.00
Seed yield, kg ha^-1^	3	137.37	4.94	67.33	2.42	0.87
Above-ground biomass, kg ha^-1^	3	1015.42	8.91	-315.33	-2.77	0.99

## Discussion

We assessed the global sensitivity of parameters across different years and regions and found that the most sensitive parameters associated with flowering and maturity dates were mostly time-related parameters representing the life cycle of rapeseed growth and the impact of temperature on growth and development. For example, EM-FL represents the time from seedling to flowering of rapeseed, whereas OPTBI represents the lowest daily temperature that does not affect flowering and development. On the other hand, soil parameters had little effect on rapeseed phenology because stage durations are mainly determined by ecotype and genotype parameters and temperature and daylength [25]. Previous sensitivity analysis studies focusing on DSSAT-CROPGRO parameters have shown that the parameters associated with phenological period were those related to time and accumulated temperature [46, 47].

Our results showed that yield, above-ground biomass, and leaf area index were very sensitive to the rate of main stem leaf emergence (TRIFL). In the CROPGRO-Canola model, the rate of main stem leaf appearance is governed by the phyllochron interval. Previous sensitivity analysis studies for the DSSAT-CERES family of models have shown that the phyllochron interval (PHINT) was one of the most sensitive crop parameters affecting winter wheat yield [48]. In addition, Li et al. [49] conducted a sensitivity analysis using different parameter ranges (±10% to ±50%) in the DSSAT-CERES model and found that above-ground biomass in winter wheat was very sensitive to PHINT. Results of this study also showed that yield and above-ground biomass was highly sensitive to some parameters associated with temperature, such as SLOBI and OPTBI, while these parameters had little effect on development rate. These results indicate that yield and above-ground biomass are affected by both length of the growth period and genetic factors. Moreover, our results showed that soil parameters had some impact on productivity indicators, such as yield and above-ground biomass, although the effect was not as high as TRIFL. This was consistent with previous studies that used the EFAST method to analyze sensitivity of soil parameters in the CERES-wheat model, and found that SDUL (drained upper limit), and SLNF (soil mineralization coefficient) to be the most sensitive soil parameters associated with wheat yield [48]. Sensitivity analyses on other crop models in CROPGRO have also shown that parameters associated with soil moisture content have a significant impact on crop yield [50].

In an actual calibration and evaluation case study, several other factors, such as grain filling duration of rapeseed, the number of seeds per pod, and weight of one thousand pods may also affect rapeseed productivity indicators. However, these parameters were not sensitive according to the results of our global sensitivity analysis. In the model, there is a trade-off between weight per seed and seed number, as well as pod size and pod number based on the daily carbon balance. Thus, the model biomass outputs were not sensitive to these parameters over the expected range used in the sensitivity analysis.

## Conclusion

The EFAST method was used to evaluate the sensitivity of the CROPGRO-Canola model biomass outputs to genetic and soil inputs. The sensitivity analysis showed that important input parameters were highly consistent spatially and temporally. The main parameters affecting growth duration in rapeseed included EM-FL (time between plant emergence and flower appearance), OPTBI (minimum daily temperature above which there is no effect on slowing normal development toward flowering), SLOBI (slope of relationship reducing progress toward flowering if TMIN for the day is less than OPTBI), RWDTH (relative width of this ecotype in comparison to the standard width per node (YVSWH), and SLAVR (specific leaf area of cultivar under standard growth conditions). The main parameters influencing yield and above-ground biomass included TRIFL (rate of appearance of leaves on the mainstem), OPTBI, SLOBI, SLAVR, and EMFL. The main parameters affecting maximum leaf area index were TRIFL, SLAVR, and EMFL. The sensitivity analysis was used to calibrate and evaluate the rapeseed model for a single location. The calibrated model gave good simulations of phenology, yield and canopy weight at maturity. Results of this study can be used to guide broader CROPGRO-Canola model evaluations with limited data in the future.
