# Correction: Sensitivity analysis of the CROPGRO-Canola model in China: A case study for rapeseed

**DOI:** 10.1371/journal.pone.0286176

**Published:** 2023-05-18

**Authors:** Mancan Xu, Chunmeng Wang, Lin Ling, William D. Batchelor, Jian Zhang, Jie Kuai

The affiliation for the third author is incorrect. Lin Ling is not affiliated with #1 but only with #2: Inspection and Quarantine Technology Communication Department, Shanghai Customs College, 201204, Shanghai, P.R. China.

Lin Ling’s email address is also incorrect. The correct email address is: flyrui0318@163.com.
